# Supplementary material for: Novel deep neural network architecture fusion to simultaneously predict short-term and long-term energy consumption
Source: PLoS One. 2025 Jan 7;20(1):e0315668. doi: 10.1371/journal.pone.0315668 (PMC11706372; doi:10.1371/journal.pone.0315668)
Supplement: Attachment — Submitted filename: Response to reviewers.pdf [file pone.0315668.s002.pdf]

## ***<u>Response to the Editor</u>***

*We are thankful to the academic editor for the encouraging and positive comments to improve the manuscript. We have revised our manuscript based on the editor's comments and highlighted them in the revised manuscript. Please, find below the referees' comments and our responses inserted after each comment in italics.*

**Editor Comments**

Please carefully revise the manuscript with two referees' comments.

***Author response:*** *We sincerely thank the academic editor for the encouraging and valuable comments. We have extensively revised the manuscript according to the two referees' comments. We hope our amendments are satisfactory and consider our current version of the manuscript for publication. Thank you.*

## *Response to the Reviewer 1*

*We are thankful to the reviewer for the encouraging and positive comments to improve the manuscript. We have revised our manuscript based on reviewer 1's comments and highlighted them in the revised manuscript. Please, find below the referees' comments and our responses inserted after each comment in italics.*

**Review Comments to the Author**
**Reviewer #1**

The manuscript addresses an important issue in energy consumption prediction by proposing a novel hybrid model that combines Convolutional Neural Networks (CNN), Long Short-Term Memory (LSTM), and Bi-directional LSTM (Bi-LSTM) architectures to predict both short-term and long-term residential energy consumption. While the approach is interesting and potentially valuable, there are several areas where the manuscript could be improved.

***Author response:*** *We sincerely thank the reviewer for the encouraging and valuable comments. We are sorry for the mistake caused by our negligence. In the revised manuscript, the mistake has been corrected and highlighted. We have tried our best to address all the concerns and comments and please find our point-by-point response in the following. We hope our amendments are satisfactory and consider our current version of the manuscript for publication.*

**Reviewer #1, Concern #1:** The paper claims to present a novel hybrid model for predicting energy consumption. However, the innovation appears to be relatively incremental given the existing literature on CNN, LSTM, and Bi-LSTM architectures.

***Author response:*** *Thank you for your thoughtful feedback. We sincerely apologize for any initial confusion and for any language that may have inadvertently introduced ambiguity. The authors greatly appreciate your efforts and insightful comments, which have been invaluable in guiding the revisions. We have extensively revised the manuscript to address these points and eliminate potential ambiguities.*

*Your observations are well-taken, and we agree with them. For the first time, we have integrated three distinct neural network layers to predict energy consumption, achieving performance superior to state-of-the-art approaches.*

*To clarify, the specific contributions and innovations of the proposed research model are outlined as follows:*

*1. Novel Layer Integration in Model Architecture:*
*While previous research has employed CNN, LSTM, and Bi-LSTM models independently, our proposed hybrid model combines these three models to predict energy consumption. This novel hybrid approach allows the model to capture both spatial and temporal features in the input data, enabling a more comprehensive analysis of energy consumption behavior that might be overlooked when using a single model.*

*2. Enhanced Feature Learning:*

*The CNN layer is positioned at the initial stage of the architecture, enabling the hybrid model to extract local patterns and outliers in energy consumption data. This approach is particularly suited to short-term energy consumption predictions, where rapid changes in consumption patterns are common. While recent studies focus primarily on LSTM and Bi-LSTM for temporal prediction, they often overlook convolutional layers for feature extraction, which our model effectively incorporates.*

*3. Bidirectional Processing with Bi-LSTM:*

*Bi-LSTM layers enhance accuracy by capturing both future and past contexts within the energy consumption time series dataset. Although rarely integrated with CNN in current research, the use of Bi-LSTM in our model provides a novel perspective to this study.*

*4. Addressing Current Constraints and Challenges:*

*The proposed model overcomes limitations commonly found in existing literature, such as the inability to generalize effectively across both short-term and long-term energy consumption predictions. Our model addresses these gaps and represents a significant addition to the existing body of knowledge on energy consumption prediction.*

*In summary, based on an extensive review of contemporary literature, while certain components of the proposed hybrid model may be present individually in previous studies, the combined use of CNN, LSTM, and Bi-LSTM for energy consumption forecasting, alongside rigorous testing on practical data, is genuinely novel. We believe these innovations merit further investigation and underscore the relevance of our study to advancing energy prediction methods and contributing to current trends.*

**Reviewer #1, Concern #2:** The rationale for combining short-term and long-term predictions in a single model is not sufficiently justified. Why is it necessary to predict both simultaneously? What specific advantages does this approach offer over using separate models for short-term and long-term predictions?

***Author response:*** *Thank you for your thoughtful feedback, and we apologize for any initial confusion. The rationale for combining short-term and long-term predictions within a single model, particularly in the context of energy consumption forecasting, is grounded in the inherent interdependencies between these temporal scales. The necessity and advantages of predicting both simultaneously are outlined below:*

*1. Interconnectedness of Short-term and Long-term Patterns*

*- Shared Influences: Short-term energy consumption patterns are often influenced by immediate factors such as weather, time of day, and operational behaviors, while long-term trends reflect seasonality, gradual demand changes, and policy-driven shifts. These patterns are not independent; rather, they are interrelated. Modeling them in isolation risks overlooking the nuanced interactions where short-term fluctuations inform long-term trends and vice versa.*

*- Comprehensive Understanding: A hybrid model that predicts both short-term and long-term consumption facilitates a holistic understanding of the data. The temporal features captured at one scale can enhance prediction accuracy at another, thereby improving the model's overall robustness.*

*- Multi-scale Feature Extraction: Concurrent application of CNN, LSTM, and Bi-LSTM networks enables the model to focus on both short-term variations and long-term changes in the operational context. CNN networks extract related spatial patterns, while LSTM and Bi-LSTM layers capture temporal sequential patterns, allowing learning to occur simultaneously across different time periods.*

*- Joint Learning: Simultaneously learning short-term and long-term patterns benefits from feedback loops, where short-term predictions inform long-term trends and vice versa. This cross-scale learning ensures better generalization, as the model adapts its predictions based on dynamically changing short-term patterns while remaining aligned with long-term features.*

*2. Addressing Volatility and Seasonal Trends*

*- Reducing Volatility: Energy consumption figures can be volatile on a short-term basis, with sudden spikes that are not always accounted for by longer-term trends. The hybrid model captures near-term irregularities that can be integrated into longer-term predictions, enhancing robustness against rapid changes.*

*- Seasonality Considerations: Long-term forecasts also account for seasonal trends and shifts in consumption behavior over time. The integration of short-term predictions enriches these longer-term projections with current data, rendering targets more responsive to real-time contexts and increasing accuracy.*

*3. Enhanced Generalization and Adaptability*

*- A unified model demonstrates superior generalization capabilities to new, unseen data compared to independent classifiers, leading to cross-generalization. This time-scale-agnostic learning capability enhances the model's adaptability to changes in short-term demand as well as long-term consumption patterns, thus improving generalization across diverse use cases.*

*- Dynamic System Design: As dynamic energy systems frequently experience changes in consumption patterns, a hybrid approach that incorporates both short-term data and long-term evolution avoids the need for retraining two separate models.*

*4. Field Applications in Energy Management*

*The practical applications of such a model in real-world energy management systems are numerous. Planning often requires both very short-term forecasts (e.g., for grid balancing) and longer-term predictions (e.g., for investment planning or policy preparation). Integrating historical path analysis with projections and evaluations of alternative paths within the same model reduces decision-making complexity and provides actionable insights across both long-term planning and immediate operational actions.*

*In conclusion, combining short- and long-term predictions into a single model offers several technical and operational advantages over multi-model approaches. First, multi-model frameworks do not effectively exploit the relationships between different temporal patterns in energy data. Second, they require additional computational resources to integrate prediction results from disparate models. Finally, performance improvements may not be realized with multi-models if there is limited similarity between short-term and long-term patterns. Other benefits of our hybrid approach include enhanced*

*understanding of shared influences, improved resource utilization efficiency, better handling of volatility and seasonal trends, and enhanced generalization and adaptability.*

**Reviewer #1, Concern #3:** The manuscript does not provide adequate details on how the data for different prediction horizons were obtained. Are these data sets collected from the same source? How do the authors handle the differences in data frequency and resolution?

***Author response:*** *Thank you for your insightful comment. Yes, the datasets utilized in our research experiments were collected from the same source.*

*To address the differences in data frequency and resolution, we implemented the following approaches:*

*1. Data Aggregation: To facilitate long-term predictions using the proposed approach, we aggregated data at the minute level. Specifically, to obtain hourly aggregated data, we calculated the sum or mean of energy consumption across all minutes within an hour. This method allows the model to capture the necessary time granularity for short-term predictions while also accommodating long-range forecasts.*

*2. Resampling Techniques: We applied resampling methods to align the data with the required prediction horizon. For hourly predictions, minute-level data were resampled to create hourly averages, ensuring that the resolution matches the forecast target. This approach safeguards against the loss of information when comparing data across different prediction horizons and ensures that significant patterns are not overlooked.*

*3. Feature Consistency: To maintain consistency across different prediction horizons, all features used in the model were normalized. This normalization ensures that features contributing to short-term predictions and those relevant to long-term predictions are on the same scale, facilitating accurate comparisons.*

*By aggregating the data and applying resampling techniques, the manuscript ensures that a single dataset can be utilized for making predictions at varying temporal resolutions. The application of resampling effectively addresses the challenges associated with frequency and resolution divergence, thereby making the model suitable for both short- and long-term predictions without the need for separate datasets.*

**Reviewer #1, Concern #4:** The manuscript lacks sufficient data visualization to support its claims. Data visualizations such as time series plots, correlation matrices, or feature importance rankings would provide valuable insights into the data and the model's performance.

***Author response:*** *Thank you for your valuable feedback. In response to your valuable suggestion, we have added a subsection titled "Data Visualization" in the revised version of the manuscript. This addition aims to enhance the reader's understanding of the data and its analysis.*

## Data Visualization

In the following Figure 2, a correlation matrix analysis is given. In the following table, positive 1 indicates there is a positive correlation, -1 indicates there is a negative correlation and 0 indicates there is no correlation, the first matrix shows the correlation of minute analysis, whereas the second matrix shows the correlation of hourly data analysis, the third matrix shows correlation matrix of daily data and last matrix shows correlation matrix of weekly data.

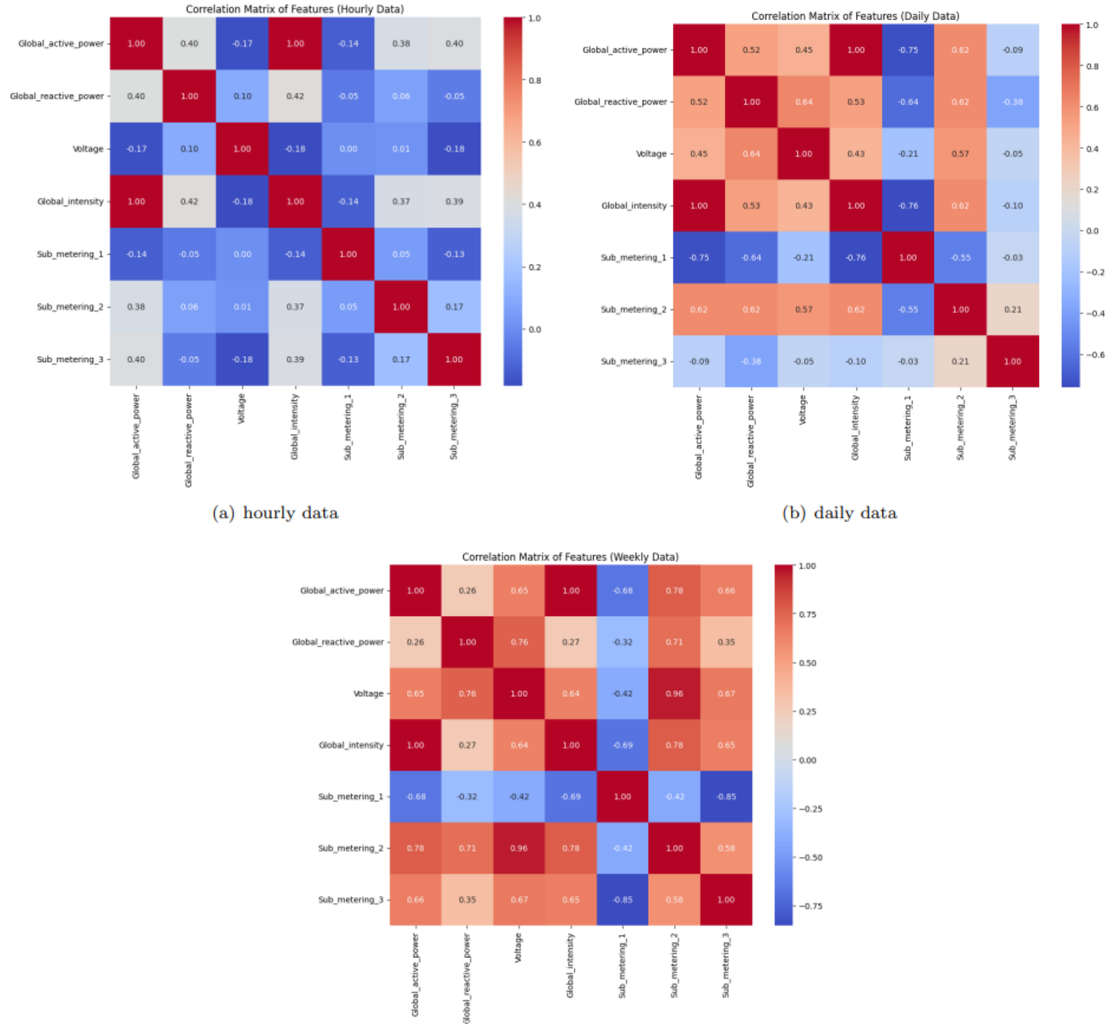


(a) hourly data (b) daily data

(c) weekly data

**Fig 2.** A correlation matrix analysis.

*We have included time series plots and feature importance rankings in the results section of the manuscript. These visualizations provide critical insights into the temporal patterns of the data and highlight the relative significance of each feature in our model's predictions.*

## Time series during training analysis

Figure 4 graphs show the model loss over Epochs. For minute analysis validation loss is less than 0.005, for hourly data validation loss is 0.002, for daily analysis validation loss is near about 0.0 and for weekly data validation loss is less than 0.05. These validation losses over Epochs show that the proposed model is not overfitting and achieving very close alignment to the validation data, hence it proves that our proposed model is working well.

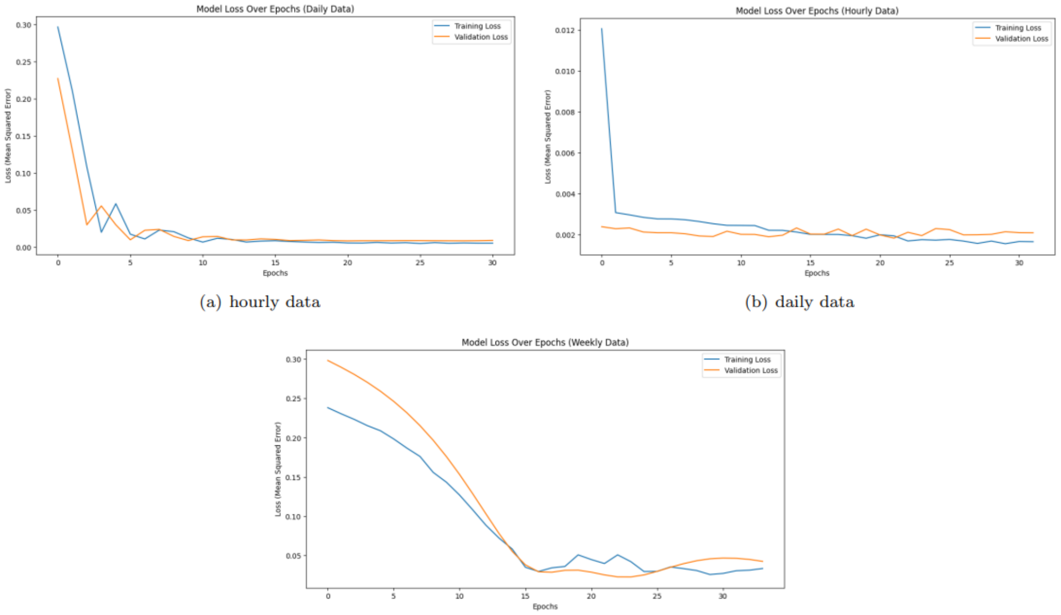


(a) hourly data (b) daily data

(c) weekly data

**Fig 4.** The Time series during training analysis.

# Feature importance rankings analysis

Figure 5 graphs show feature importance ranking in the dataset. For minute data global_active_power has a great influence on the dataset and it contributes heavily to predicting with model due to its high correlation with prediction. For other analyses such as hourly data, daily data and weekly data, sub_metering_3 has high importance due to its predictive patterns and high correlation with target.

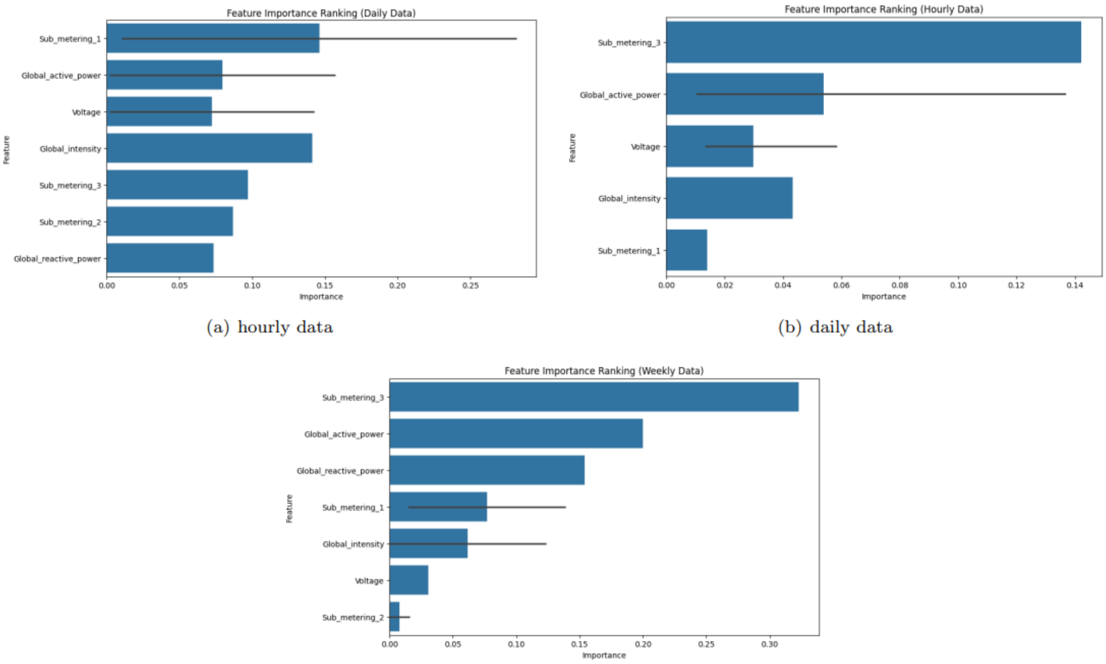


(a) hourly data

(b) daily data

(c) weekly data

**Fig 5.** The Feature importance rankings analysis.

**Reviewer #1, Concern #5:** While the manuscript reports performance metrics such as Mean Square Error (MSE) and Mean Absolute Error (MAE), it does not provide a comprehensive comparison with baseline models or other state-of-the-art methods.

***Author response:*** *Thank you for your thoughtful comment. In accordance with your valuable suggestion, we have added a subsection titled "Performance Summarization with Baseline Models" in the revised version of the manuscript. This subsection provides a comprehensive comparison of our model's performance against established baseline models, highlighting the improvements and effectiveness of our proposed approach.*

### Performance summarization with baseline models

Table 8 summarizes the average performance of the proposed model compared to similar techniques in the literature. The values are computed by averaging minute, hourly, daily and weekly results. Across these intervals, the proposed model consistently outperforms others based on four standard evaluation measures of MSE, RMSE, MAE and MAPE. This superiority extends to minute, hourly, daily and weekly data from the IHEPC



dataset. This work can enhance intelligent power management systems, especially in predicting future electric power consumption. The average results are visualized in Figure 6.

**Table 8.** Average performance of the proposed model compared to with baseline models.

| #No | Model | MSE | RMSE | MAE | MAPE |
|---|---|---|---|---|---|
| 1 | Linear Regression [4] | 0.30775 | 0.544 | 0.408 | 63.07 |
| 2 | LSTM [4] | 0.40225 | 0.59925 | 0.45275 | 42.58 |
| 3 | CNN-LSTM [2] | 0.232 | 0.4595 | 0.294 | 32.835 |
| 4 | EECP-CBL [4] | 0.11575 | 0.3115 | 0.2145 | 25.545 |
| 5 | Decision tree [11] | 0.59 | 0.77 | 0.54 | - |
| 6 | SVR [11] | 0.59 | 0.77 | 0.49 | - |
| 7 | CNN [11] | 0.37 | 0.67 | 0.47 | - |
| 8 | CNN-GRU [11] | 0.22 | 0.47 | 0.33 | - |
| 9 | **Proposed Model** | 0.00035 | 0.01825 | 0.0057 | 24.1125 |

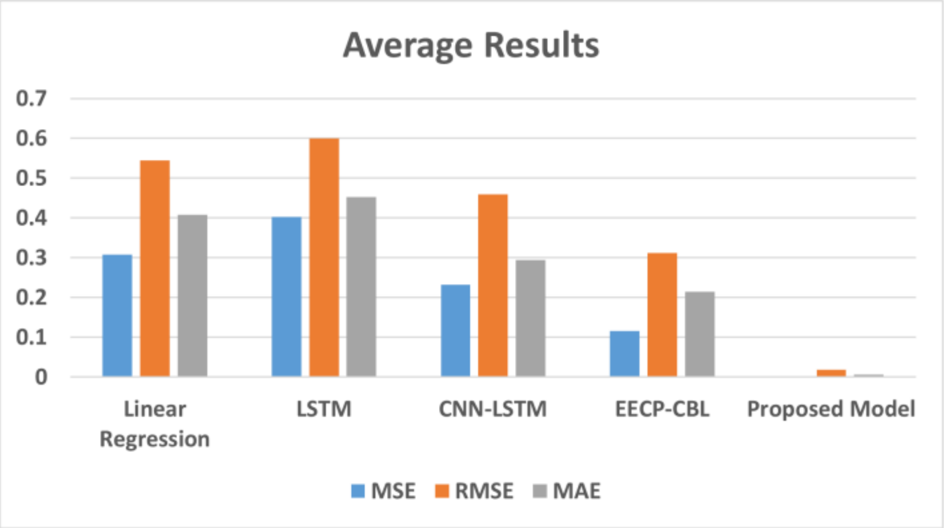


**Fig 6.** Average performance of the proposed model compared to similar techniques.

**Reviewer #1, Concern #6:** The evaluation section would benefit from additional details about the experimental setup, including the size of the training and testing datasets, the hyper parameters used for the models, and the computational resources required. Multiple experiments are also necessary.

***Author response:*** *We greatly appreciate your insightful suggestion and sincerely apologize for any inconvenience caused by the previous ambiguous language. In accordance with your valuable feedback, we have included more explicit details regarding the experimental setup in the revised version of the manuscript. This addition aims to enhance clarity and provide a comprehensive understanding of our methodology.*

## Experimental Setup

The research experiments are conducted using Google Colab, and the computations are performed through freely available GPUs on the Google Colab platform. For training and testing sets, we use a 70:30 split ratio. In detail, 70% of the dataset is set out for training the model, whereas the rest 30% is utilized for testing and validation. In addition, 5-fold cross-validation is practiced to ensure the robustness of the proposed model. The model is implemented using Keras with TensorFlow as the backend, utilizing Python for data processing and model training. The hyperparameters used for the proposed models are expressed in Table 2.

**Table 2.** The hyperparameters used for the models.

| **Models** | **Hyperparameters Description** |
|---|---|
| CNN | Number of filters: 64, Kernel size: 3, Activation function: ReLU, Pooling size: 2, Dropout rate: 0.2 |
| LSTM | Number of units: 128, Return sequences: True for the first LSTM layer, False for the second |
| Bi-LSTM | Number of units: 128, Optimizer: The Adam optimizer was chosen due to its adaptive learning rate feature for faster convergence. Loss Function: The MSE was selected as the loss function to find the best JSON model. Batch Size: 32, Epochs: The model was trained for 100 epochs, with early stopping applied based on the validation loss (patience of 10 epochs). |

**Reviewer #1, Concern #7:** Ensure that all acronyms and technical terms are clearly defined when first introduced to readers.

***Author response:*** *Thank you for your thoughtful note. In accordance with your valuable suggestion, we have added a subsection titled "Acronyms" in the revised version of the manuscript. This subsection provides definitions and explanations for all acronyms used throughout the document, thereby enhancing clarity and ensuring that readers can easily follow the content.*

## Acronyms

The acronyms in Table 10 are provided for clarity and convenience. These acronyms are intended to facilitate clear communication in the discussion of the proposed model, related work, and evaluation metrics.

**Table 10.** Description of acronyms used in this manuscript.

| Short Name | Abbreviation |
|---|---|
| EECP | Electric Energy Consumption Prediction |
| CNN | Convolutional Neural Network |
| Bi-LSTM | Bi-directional Long Short-Term Memory |
| IHEPC | Individual Household Electric Power Consumption dataset |
| LSTM | Long Short-Term Memory |
| RNN | Recurrent Neural Network |
| MSE | Mean Square Error |
| RMSE | Root Mean Square Error |
| MAE | Mean Absolute Error |
| MAPE | Mean Absolute Percentage Error |
| MLP | Multilayer Perceptron |
| ReLU | Rectified Linear Unit |
| EECP-CBL | Electric Energy Consumption Prediction model utilizing the combination of CNN and Bi-LSTM |
| RF | Random forest |
| RT | Random Tree |
| CFS | Correlation-based feature selection |
| NCA FS | Neighborhood Component Analysis Regression-based feature selection |
| MRE | Mean relative error |
| SMAPE | Symmetric mean absolute percentage error |
| EDA | Exploratory data analysis |
| IMFs | Intrinsic mode functions |
| MLR | Multiple liner regression |
| BPNN | Back-propagation neural networks |
| EDL | Extreme deep learning |
| GBR | Gradient boosting regression |
| IDT | Information diffusion technology |
| HMTD | Heuristic mega-trend diffusion |
| LSTM-AE | Auto encoder long short-term memory |
| GRU | Gated-recurrent unit |
| SD | Standard deviation |
| EMD | Empirical mode decompositions |
| FFANN | Feedforward artificial neural network |

***Author's final remark:*** *Thank you for your thoughtful and constructive feedback, which has been invaluable in refining our manuscript. We believe our findings will be of significant interest to the community, and we have meticulously addressed your comments, making revisions to enhance both the clarity and quality of our work.*

*We respectfully submit the revised version for your consideration for publication in* ***PLOS ONE****. We sincerely appreciate your time and dedication in reviewing our paper and welcome any further feedback*

*or suggestions you may have. Please do not hesitate to reach out with any additional questions or recommendations.*

*Once again, thank you for your valuable insights and guidance.*

# *Response to the Reviewer 2*

*We are thankful to the reviewer for the encouraging and positive comments to improve the manuscript. We have revised our manuscript based on reviewer 2's comments and highlighted them in the revised manuscript. Please, find below the referees' comments and our responses inserted after each comment in italics.*

**Review Comments to the Author**
**Reviewer #2**

The manuscript presents a hybrid deep learning model integrating Convolutional Neural Networks (CNN), Long Short-Term Memory (LSTM), and Bi-directional LSTM (Bi-LSTM) for forecasting energy consumption. I have several suggestions and concerns that need to be addressed before the manuscript can be considered for publication.

***Author response:*** *We are sincerely thankful to the reviewer for the encouraging and valuable comments. We are sorry for the mistake caused by our negligence. In the revised manuscript, the mistake has been corrected and highlighted. We have tried our best to address all the concerns and comments and please find our point-by-point response in the following. We hope that our amendments are satisfactory and consider our current version of the manuscript for publication.*

**Reviewer #2, Concern #1:** Comparison with Decomposition Algorithms: The authors mention the use of Empirical Mode Decomposition (EEMD) in existing literature for daily energy consumption prediction. It is suggested that the manuscript would benefit from the inclusion of comparative experiments that analyze the performance of the proposed model against models incorporating decomposition algorithms like EMD, EEMD and CEEMDAN.

***Author response:*** *The authors sincerely appreciate your efforts and insightful comments. We apologize for any inconvenience caused by the previous ambiguous language. The manuscript has been extensively revised to address these issues.*

*In accordance with your valuable suggestion, we have added a comparison of our results with Empirical Mode Decomposition (EMD), Ensemble Empirical Mode Decomposition (EEMD), and Complete Ensemble Empirical Mode Decomposition with Adaptive Noise (CEEMDAN) in the revised version of the manuscript.*

## Comparison with Decomposition Algorithms

Table 7 shows the performance of the proposed model against models incorporating decomposition algorithms, which include EMD, EEMD, and CEEMDAN. The analysis infers that using each algorithm-propped model achieved optimal performance scores for predicting energy consumption with a minimum error rate.

**Table 7.** The comparison results with decomposition algorithms.

| Model | Minutely | | Hourly | | Daily | | Weekly | |
|---|---|---|---|---|---|---|---|---|
| EMD | MSE | 0.9694 | MSE | 0.2946 | MSE | 9.68 | MSE | 0.0016 |
| | RMSE | 0.9846 | RMSE | 0.5428 | RMSE | 0.0098 | RMSE | 0.0408 |
| | MAE | 0.7029 | MAE | 0.4162 | MAE | 0.0098 | MAE | 0.0340 |
| | MAPE | 6670.6% | MAPE | 3955.9% | MAPE | 94.38% | MAPE | 334.2% |
| EEMD | MSE | 0.9694 | MSE | 0.2946 | MSE | 0.2017 | MSE | 0.3093 |
| | RMSE | 0.9846 | RMSE | 0.5428 | RMSE | 0.4492 | RMSE | 0.5561 |
| | MAE | 0.7029 | MAE | 0.4162 | MAE | 0.3637 | MAE | 0.4427 |
| | MAPE | 6670.6% | MAPE | 3955.9% | MAPE | 3537.3% | MAPE | 4200.2% |
| CEEMDAN | MSE | 1.0046 | MSE | 1.0081 | MSE | 1.0136 | MSE | 1.0043 |
| | RMSE | 1.0023 | RMSE | 1.0040 | RMSE | 1.0068 | RMSE | 1.0021 |
| | MAE | 0.9004 | MAE | 0.9030 | MAE | 0.9005 | MAE | 0.9003 |
| | MAPE | 9691.6% | MAPE | 9401.7% | MAPE | 8675.6% | MAPE | 8762.2% |

**Reviewer #2, Concern #2:** Theoretical Justification: The manuscript claims that the proposed model can effectively handle both short-term and long-term energy consumption predictions. However, a theoretical explanation is lacking on how the model achieves this dual capability. The authors should elaborate on the theoretical aspects and mechanisms that allow the model to address the complexities of short-term and long-term predictions simultaneously.

***Author response:*** *Thank you for your valuable suggestions. In response, we have provided a theoretical justification for how the proposed hybrid model achieves dual capability in both short-term and long-term energy consumption predictions:*

*The hybrid model integrates Convolutional Neural Networks (CNN), Long Short-Term Memory (LSTM) networks, and Bidirectional LSTM (Bi-LSTM) networks, leveraging their complementary capabilities to facilitate accurate forecasting. The following points elucidate the theory behind its effectiveness in addressing the complexities of short- and long-term forecasting:*

*1. Short-term Feature Extraction using Convolutional Neural Networks (CNN)*

*CNNs are particularly effective for energy consumption prediction due to their ability to extract meaningful localized features, such as sudden fluctuations or anomalies at minute or hourly intervals. The CNN layer filters out noisy data and identifies irregular trends, a common issue in short-term energy consumption data. By focusing on relevant patterns and providing clear outputs to subsequent layers, CNNs are ideal for short-term energy consumption predictions.*

*2. LSTM for Sequential Learning*

*LSTMs are designed to process sequential data with long dependencies, making them essential for time series energy consumption data. With their memory cells and gating mechanisms (forget gate, input gate, and output gate), LSTMs can retain information over extended periods. Additionally, LSTMs effectively address the vanishing gradient problem that traditional Recurrent Neural Networks (RNNs) often encounter. This capability makes LSTMs well-suited for modeling trends and dependencies that extend over longer time horizons, such as daily or weekly energy consumption predictions.*

*3. Contextual Understanding using Bidirectional LSTM (Bi-LSTM)*

*Bi-LSTMs enhance sequence learning by processing input in both forward and backward directions. This allows the model to access information from both previous and future states simultaneously, which is particularly useful for long-term predictions. By capturing historical trends leading up to the present and forecasting future consumption behavior based on these trends, Bi-LSTMs improve the understanding of the overall context within which short-term fluctuations occur. This dual perspective enhances the accuracy of long-term consumption predictions, as future trends are integrated even when making short-term forecasts.*

*4. Theoretical Synergy of CNN, LSTM, and Bi-LSTM in Hybrid Models*

*The proposed hybrid model employs CNN for extracting local, short-term patterns, while LSTM and Bi-LSTM capture global, long-term dependencies. This creates a theoretical synergy: the CNN identifies fine-grained changes in minute-by-minute consumption variability, whereas LSTM and Bi-LSTM grasp broader temporal patterns, such as seasonal or weekly consumption trends. Energy consumption data exhibits multi-scale dynamics, where short-term fluctuations are embedded within long-term trends. Thus, the hybrid model effectively addresses this complexity by utilizing CNN to capture high-frequency, short-term features, alongside LSTM and Bi-LSTM for low-frequency, long-term dependencies. This enables the model to predict short-term spikes and long-term trends concurrently through multi-scale learning.*

*In conclusion, the hybrid model effectively addresses both short-term and long-term predictions due to the complementary capabilities of CNNs, LSTMs, and Bi-LSTMs. CNNs extract localized short-term patterns, while LSTMs and Bi-LSTMs capture long-term temporal dependencies and bidirectional context. This multi-scale feature extraction and sequence learning mechanism allows the model to generate credible forecast results across various time horizons. Additionally, it improves performance and reduces computational overhead by integrating short-term and long-term predictions within a unified framework, thereby enhancing adaptability in dynamic environments.*

**Reviewer #2, Concern #3:** Rationale for Model Parameter Selection: Please provide the basis for the hyperparameters chosen for the models.

***Author response:*** *Thank you for your valuable suggestions. For the proposed hybrid model integrating Convolutional Neural Networks (CNN), Long Short-Term Memory (LSTM), and Bidirectional LSTM (Bi-LSTM), the selection of hyperparameters is based on the following rationale:*

*The most suitable hyperparameters are identified through iterative training and validation processes conducted during the experimental phase. Specifically, we utilized a recursive approach involving training and testing, employing a k-fold cross-validation mechanism to ensure robust evaluation of model performance.*

*In addition to this empirical method, we considered theoretical foundations alongside our experimental experience. The hyperparameters for the hybrid model of CNN, LSTM, and Bi-LSTM were selected based on theoretical principles, such as the ability of CNNs to perform effective feature extraction and the capability of LSTMs to preserve temporal dependencies. This selection was further refined through empirical tuning, informed by extensive experimentation.*

**Reviewer #2, Concern #4:** Ablation Study: To better understand the contribution of each component of the proposed model, an ablation study could be beneficial. The authors are encouraged to compare the performance of the model with different configurations, such as CNN+BiLSTM, CNN+LSTM, and BiLSTM+LSTM.

***Author response:*** *Thank you for your valuable suggestions. In accordance with your recommendation, we have conducted an ablation study to analyze the contribution of each component in the proposed model. The revised manuscript now includes a comparison among the configurations of CNN + LSTM, CNN + Bi-LSTM, and Bi-LSTM + LSTM. This analysis highlights the individual contributions of each component and provides insights into their respective impacts on model performance.*

## Ablation Study

In our ablation study, we conducted a comparative analysis to investigate the performance of hybrid deep learning models. The results, as detailed in Table 9 for each model. The CNN-LSTM model yielded an MSE of 0.095, RMSE of 0.309, MAE of 0.238, and MAPE of 31.84%, indicating a reasonable predictive performance. However, the CNN+Bi-LSTM model, with slightly higher error metrics, did not significantly outperform the CNN-LSTM. Notably, the BiLSTM+LSTM configuration achieved superior accuracy, with minimal errors across all metrics. This significant reduction in errors suggests that BiLSTM+LSTM is highly effective for this task, capturing complex patterns more accurately compared to the other configurations.

**Table 9.** The ablation study analysis.

| Model | MSE | RMSE | MAE | MAPE |
|---|---|---|---|---|
| CNN-LSTM | 0.095 | 0.309 | 0.238 | 31.84 |
| CNN+Bi-LSTM | 0.104 | 0.322 | 0.257 | 31.83 |
| BiLSTM+LSTM | 0.008 | 0.090 | 0.067 | 11.51 |

**Reviewer #2, Concern #5:** Figure 3 Clarifications and Enhancements: The visualization in Figure 3 is not clear and uses a mix of bar charts and line graphs without a clear rationale. The authors should reconsider the design of Figure 3 to ensure that it effectively communicates the results. Additionally, the figure should be redrawn with higher resolution to improve clarity.

***Author response:*** *Thank you for your valuable suggestions. In accordance with your feedback, we have revised what was previously Figure 3, which is now designated as Figure 6 in the updated manuscript. The modifications enhance clarity and better illustrate the key concepts presented.*

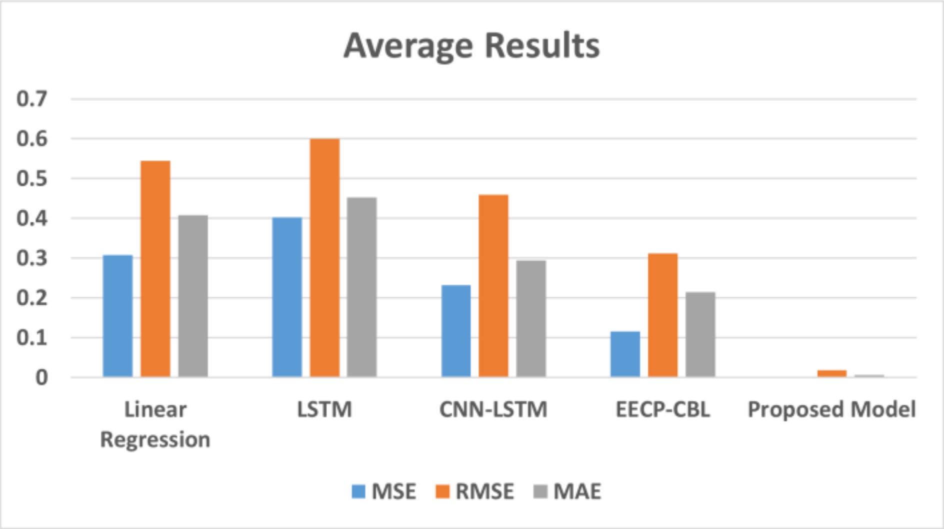


**Fig 6.** Average performance of the proposed model compared to similar techniques.

**Reviewer #2, Concern #6:** Consistency in Comparative Models: There is an inconsistency in the models compared across Table 3, Table 4, and Table 5. The authors should provide a rationale for these differences in the comparison models.

***Author response:*** *Thank you for your valuable suggestions. You are correct in your observation regarding the differences in the comparative evaluation parameters of models across Tables 3, 4, and 5; these differences were intentional and aligned with the specific objectives of each type of performance analysis.*

- *Table 3 focuses on benchmarking baseline models commonly used for the given task, providing a foundation for understanding traditional performance standards.*
- *Table 4 introduces more advanced models, specifically incorporating hybrid architectures that have demonstrated promising results in recent studies. This allows us to observe how these architectures influence predictive accuracy and error metrics.*
- *Table 5 evaluates models with various configurations and parameter adjustments, specifically assessing the contribution of each model component.*

*This structured approach facilitates a detailed, stepwise evaluation of model improvements across multiple architectures, thereby providing a clearer understanding of model efficacy under different conditions and evaluation parameters.*

***Author's final remark:*** *Thank you for your thoughtful and constructive feedback, which has been invaluable in refining our manuscript. We believe our findings will be of significant interest to the community, and we have meticulously addressed your comments, making revisions to enhance both the clarity and quality of our work.*

*We respectfully submit the revised version for your consideration for publication in **PLOS ONE**. We sincerely appreciate your time and dedication in reviewing our paper and welcome any further feedback or suggestions you may have. Please do not hesitate to reach out with any additional questions or recommendations.*

*Once again, thank you for your valuable insights and guidance.*
