# Supplementary material for: Novel deep neural network architecture fusion to simultaneously predict short-term and long-term energy consumption
Source: PLoS One. 2025 Jan 7;20(1):e0315668. doi: 10.1371/journal.pone.0315668 (PMC11706372; doi:10.1371/journal.pone.0315668)
Supplement: Attachment — Submitted filename: R2-Response to reviwer comments-Plos One.pdf [file pone.0315668.s003.pdf]

Response to Comments

Manuscript ID: PONE-D-24-30954R1

Title: Novel Deep Neural Network Architecture Fusion to Simultaneously Predict Short-Term and Long-Term Energy Consumption

Dear Editor,

Thank you very much for allowing us to revise the manuscript. We would like to thank the editor and all the reviewers for their valuable comments and suggestions. Based on the feedback, we have revised our manuscript. The detailed modifications to address reviewers' comments are provided in the following. For clarity, we have marked our responses in blue. Whenever we update a paragraph in the manuscript, we mark it as a red color.

Reviewer #1

Point 1. The abstract is overly verbose, with much of it spent summarizing previous literature rather than succinctly presenting the motivation and unique contributions of the research. The novelty is not clearly convincing, making it difficult for readers to grasp the specific advancements introduced.

Response: The authors are highly grateful for your efforts and insightful comments. We apologize for the inconvenience and inappropriate language that raised ambiguity. The paper is extensively revised to remove ambiguity.

As per your valuable suggestion, we have revised the abstract in the updated version of the manuscript as:

"Energy is integral to the socio-economic development of every country. This development leads to a rapid increase in the demand for energy consumption. However, due to the constraints and costs associated with energy generation resources, it has become crucial for both energy generation companies and consumers to predict energy consumption well in advance. Forecasting energy needs through accurate predictions enables companies and customers to make informed decisions, enhancing the efficiency of both energy generation and consumption. In this context, energy generation companies and consumers seek a model capable of forecasting energy consumption both in the short term and the long term. Traditional models for energy prediction focus on either short-term or long-term accuracy, often failing to optimize both simultaneously. Therefore, this research proposes a novel hybrid model employing Convolutional Neural Network (CNN), Long Short-Term Memory (LSTM), and Bi-directional LSTM (Bi-LSTM) to simultaneously predict both short-term and long-term residential energy consumption with enhanced accuracy measures. The proposed model is capable of capturing complex temporal and spatial features to predict short-term and long-term energy consumption. CNNs discover patterns in data, LSTM identifies long-term dependencies and sequential patterns and Bi-LSTM identifies complex temporal relations within the data. Experimental evaluations expressed that the proposed model outperformed with a minimum Mean Square Error (MSE) of 0.00035 and Mean Absolute Error (MAE) of 0.0057. Additionally, the proposed hybrid model is compared with existing state-of-the-art models, demonstrating its superior performance in both short-term and long-term energy consumption predictions."

Point 2. The paper lacks logical coherence; for example, the dataset is introduced within the methodology section, which is misplaced. Additionally, the research motivation is presented only after the literature review and formulation of the research question, creating a confusing sequence. Reorganizing the content would improve clarity.

Response: We again apologize for the inconvenience and inappropriate language that raised ambiguity. As per your valuable suggestion, we have reorganized the content in the updated version of the manuscript.

Point 3. The order of the three models (CNN, LSTM, and Bi-LSTM) in the hybrid model lacks justification. An explanation for this specific order, as well as a consideration of alternative arrangements, would strengthen the methodology.

Response: As per your valuable suggestion we have provided the justification regarding the order of the three models (CNN, LSTM, and Bi-LSTM) as:

For the first time, we have combined the three neural network layers (CNN, LSTM, and Bi-LSTM) for predicting energy consumption and achieved high performance compared to state-of-the-art studies.

In addition, the specific justification regarding the order of the three models is highlighted in the following section:

1. Novel Layers fusion of model's architectures:

   Recent research separately used CNN, LSTM, and Bi-LSTM models, where our proposed hybrid model integrates three models for energy consumption prediction. This unique hybridization allows proposed model to capture spatial and temporal features of input data which make it possible to handle energy consumption behavior from different perspectives that might be ignored when a single model is employed.

2. Enhanced Feature Learning:

   CNN layer is deployed firstly in the architecture which allow hybrid model to extract local patterns and outliers in energy consumption data. This is usually most suitable for very short-term energy consumption prediction where the consumption pattern may quickly change. Recent studies focus on LSTM and Bi_LSTM for temporal prediction and neglecting the convolutional layers in feature extractions.

3. Bidirectional Processing:

   Bi-LSTM is important to improve accuracy and it has ability to capture both future and past contexts in the energy consumption time series dataset. In recent studies very rarely Bi-LSTM is integrated with CNN which provides a novel angel to our study.

3. Tackling Present Constraints and Other Obstacles:

   Proposed model overcome the limitations in existing literature such as inabilities to generalize across short-term and long-term energy consumption prediction simultaneously. Proposed model fills these gaps up contributes toward the existing body of knowledge in energy consumption prediction as a considerable addition.

In summary, according to the appraisal of the available contemporary resources on the subject, even though the proposed novel hybrid model features several components present in the literature, the combined use of CNN, LSTM, and Bi-LSTM for the task of energy consumption forecasting and its thorough testing on practical data is genuinely new and extends its usefulness within real-world situations. We feel these revolutions ought to be looked into more thoroughly and stress

upon the relevance of our study towards the improvement of energy prediction methods and their relevance to the existing trends.

Point 4. The figures are low quality, with inconsistent font sizes, and they lack essential comparative elements, such as prediction comparison curves, which would be more informative than the current error convergence graphs. Adding these would improve the visual relevance and readability.

Response: As per your valuable suggestion we have revised the figures in the updated version of manuscript. In addition, we have added the prediction comparison curves as:

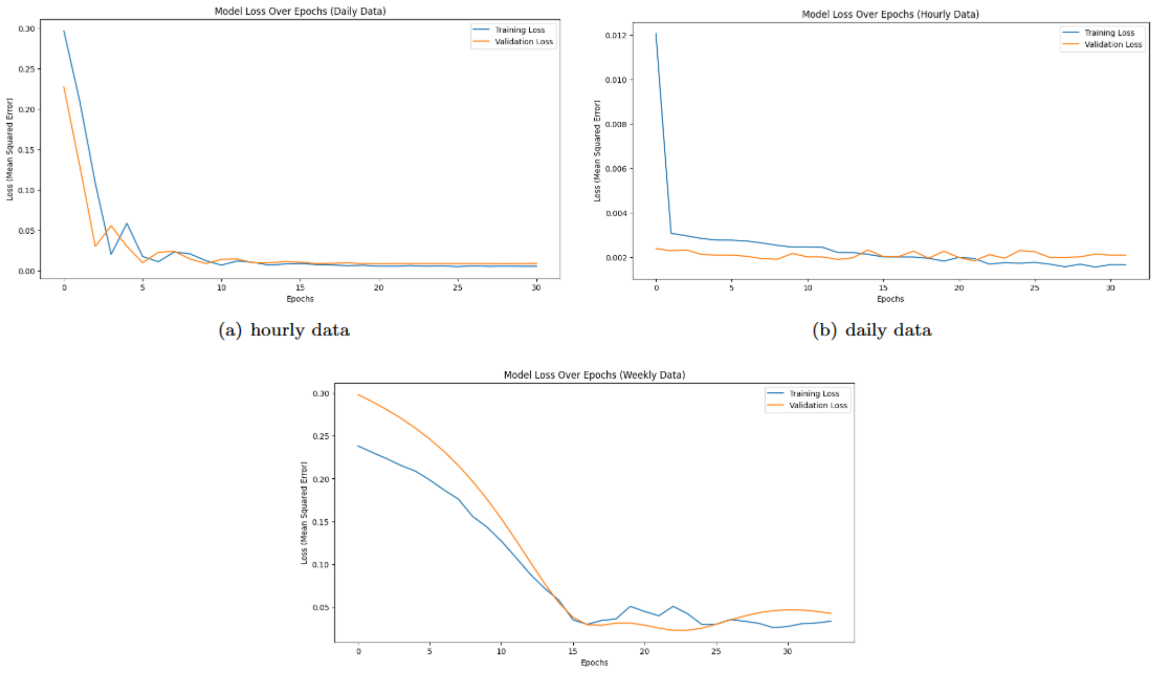

(a) hourly data

(b) daily data

(c) weekly data

Fig 4. The Time series during training analysis.

Point 5. Lastly, the English language quality is poor, with significant grammatical and syntactical issues throughout. Professional editing or proofreading is strongly recommended to enhance the readability and professionalism of the manuscript.

Response: We again apologize for the inconvenience and inappropriate language that raised ambiguity. The paper is extensively revised to remove ambiguity.

Reviewer #3

Thank you for the opportunity to review this article. Overall, I believe the topic discussed in this paper is very meaningful, and the structure is fairly clear. The model proposed by the authors effectively addresses both long-term and short-term energy consumption issues, which holds significant value for time-series forecasting problems. Below are some of my personal comments for reference:

Point 1. The evaluation results are confusing: It appears that different models were used when testing with data of varying levels, which is somewhat perplexing. Typically, it is expected to use the same set of models for comparison.

Response: The authors are highly grateful for your efforts and insightful comments. We apologize for the inconvenience and inappropriate language that raised ambiguity. The paper is extensively revised to remove ambiguity.

We appreciate your observation regarding the evaluation process. To clarify, while it may appear that different models were used for testing at varying levels, we assure you that the same dataset, the IHEPC dataset, spanning a four-year period (2006–2010) and comprising 2,075,259 records with 12 attributes, was consistently used throughout our research.

Our approach involved evaluating the proposed model across three distinct temporal granularities hourly, daily, and monthly data. This was done to thoroughly assess the model's adaptability and performance at various levels of aggregation, which is crucial for understanding energy consumption patterns in diverse scenarios.

Point 2. The model comparison is not comprehensive enough: The proposed model combines CNN, LSTM, and Bi-LSTM. As part of the ablation study, adding evaluations of Bi-LSTM would further validate the significance of each component in the proposed model architecture. Additionally, the attention mechanism is widely used in time series prediction tasks, and readers would be interested in a performance comparison between the proposed method and attention-based models.

Response: As per your valuable suggestion we have revised the ablation study and added the attention-based model's comparisons in the updated version of the manuscript as:

"Ablation Study

In our ablation study, we conducted a comparative analysis to investigate the performance of hybrid deep learning models. The results, as detailed in Table 9 for each model. The CNN-LSTM model yielded an MSE of 0.095, RMSE of 0.309, MAE of 0.238, and MAPE of 31.84\%, indicating a reasonable predictive performance. However, the CNN+Bi-LSTM model, with slightly higher error metrics, did not significantly outperform the CNN-LSTM. Notably, the BiLSTM+LSTM configuration achieved superior accuracy, with minimal errors across all metrics. This significant reduction in errors suggests that BiLSTM+LSTM is highly effective for this task, capturing complex patterns more accurately compared to the other configurations."

Table 9. The ablation study analysis.

Model	MSE	RMSE	MAE	MAPE
CNN-LSTM	0.095	0.309	0.238	31.84
CNN+Bi-LSTM	0.104	0.322	0.257	31.83
BiLSTM+LSTM	0.008	0.090	0.067	11.51

Attention-based model's comparisons:

"Performance summarization with baseline models

Table 8 summarizes the average performance of the proposed model compared to similar techniques in the literature. The values are computed by averaging minute, hourly, daily and weekly results. Across these intervals, the proposed model consistently outperforms others based on four standard evaluation measures of MSE, RMSE, MAE and MAPE. This superiority extends to minute, hourly, daily and weekly data from the IHEPC dataset. This work can enhance intelligent power management systems, especially in predicting future electric power consumption. The average results are visualized in Figure 6."

Table 8. Average performance of the proposed model compared to with baseline models.

#No	Model	MSE	RMSE	MAE	MAPE
1	Linear Regression [4]	0.30775	0.544	0.408	63.07
2	LSTM-attention layer [4]	0.40225	0.59925	0.45275	42.58
3	CNN-LSTM [2]	0.232	0.4595	0.294	32.835
4	EECP-CBL [4]	0.11575	0.3115	0.2145	25.545
5	Decision tree [11]	0.59	0.77	0.54	-
6	SVR [11]	0.59	0.77	0.49	-
7	CNN-attention layer [11]	0.37	0.67	0.47	-
8	CNN-GRU [11]	0.22	0.47	0.33	-
9	**Proposed Model**	0.00035	0.01825	0.0057	24.1125

Point 3. Flaws in the design of the comparative experiment: the authors considered that their proposed model performed well in both long-term and short-term forecasting. However, the experimental design appeared to have only adjusted the time granularity without changing the sequence length, which could not intuitively demonstrate the model's effectiveness on both long and short sequences.

Response: We acknowledge the concern regarding the design of the comparative experiment. While we adjusted the time granularity to evaluate the model's performance across different forecasting horizons, we recognize that varying sequence lengths could provide a more comprehensive assessment of its effectiveness. In response, we have revised the experimental setup to include evaluations using both short and long input sequences, ensuring a clearer demonstration of the model's capability in handling diverse sequence lengths for both long-term and short-term forecasting. We appreciate your insightful comment and have incorporated this improvement in the revised manuscript.

Point 4. The description of the ablation experiments is not sufficiently clear: the paper used data at different levels, but it was not explicitly stated in the ablation experiment whether the data used corresponds to a specific level or was an aggregated result across different levels. Additionally, including the baseline results in Table 9 would facilitate easier comparison.

Response: As per your valuable suggestion we have revised the description of the ablation experiments in the updated version of the manuscript as:

"Ablation Study

In our ablation study, we conducted a comparative analysis to investigate the performance of hybrid deep learning models using a complete dataset. The results, as detailed in Table 9 for each model. The CNN-LSTM model yielded an MSE of 0.095, RMSE of 0.309, MAE of 0.238, and MAPE of 31.84\%, indicating a reasonable predictive performance. However, the CNN+Bi-LSTM model, with slightly higher error metrics, did not significantly outperform the CNN-LSTM. Notably, the BiLSTM+LSTM configuration achieved superior accuracy, with minimal errors across all metrics. This significant reduction in errors suggests that BiLSTM+LSTM is highly effective for this task, capturing complex patterns more accurately compared to the other configurations."

Table 10. The ablation study analysis.

Model	MSE	RMSE	MAE	MAPE
CNN-LSTM	0.095	0.309	0.238	31.84
CNN+Bi-LSTM	0.104	0.322	0.257	31.83
BiLSTM+LSTM	0.008	0.090	0.067	11.51

Point 5. Limited discussion on computational complexity: The authors didn't discuss the computational complexity of their proposed model, which was crucial for evaluating its scalability and practical use in real-world applications.

Response: As per your valuable suggestion we have added the computational complexity in the updated version of the manuscript as:

"Computational complexity

The computational complexity of the proposed model in Table 9 demonstrates its efficiency across varying temporal granularities. Using the IHEPC dataset, the model required 486 seconds for daily data, 119 seconds for weekly data, and just 32 seconds for hourly data. This performance highlights the model's scalability and suitability for both coarse-grained and fine-grained energy consumption analysis, ensuring practical usability across diverse applications."

Table 9. The computational complexity of the proposed model.

Data Portions	Runtime Computations (Seconds)
For Daily Data	486s
For Weekly Data	119s
For Hourly Data	32s

Point 6. The results in the charts are not sufficiently clear: the font in the images is too small. What does the black line in Figure 5 represent? Why are only some features labeled?

Response: The black line in Figure 5 represents the error values. Since this graph is based on feature importance, we have selected the most important features and visualized their importance values.

Recommendations:

Point 1. In evaluations based on data of different levels, try to use only the same type of models. Include the performance of the baseline model Bi-LSTM as well as attention-based models.

Response: As per your valuable suggestion we have included the performance of the baseline model Bi-LSTM as well as attention-based models in the updated version of the manuscript as:

"Performance summarization with baseline models

Table 8 summarizes the average performance of the proposed model compared to similar techniques in the literature. The values are computed by averaging minute, hourly, daily and weekly results. Across these intervals, the proposed model consistently outperforms others based on four standard evaluation measures of MSE, RMSE, MAE and MAPE. This superiority extends to minute, hourly, daily and weekly data from the IHEPC dataset. This work can enhance intelligent power management systems, especially in predicting future electric power consumption. The average results are visualized in Figure 6."

Table 8. Average performance of the proposed model compared to with baseline models.

#No	Model	MSE	RMSE	MAE	MAPE
1	Linear Regression [4]	0.30775	0.544	0.408	63.07
2	LSTM-attention layer [4]	0.40225	0.59925	0.45275	42.58
3	CNN-LSTM [2]	0.232	0.4595	0.294	32.835
4	EECP-CBL [4]	0.11575	0.3115	0.2145	25.545
5	Decision tree [11]	0.59	0.77	0.54	-
6	SVR [11]	0.59	0.77	0.49	-
7	CNN-attention layer [11]	0.37	0.67	0.47	-
8	CNN-GRU [11]	0.22	0.47	0.33	-
9	Bi-LSTM [3]	0.104	0.322	0.257	31.83
10	**Proposed Model**	0.00035	0.01825	0.0057	24.1125

Point 2. Adding an evaluation of the model's predictive performance across different sequence lengths could better demonstrate the robustness of the proposed model.

Response: As per your valuable suggestion we have revised the evaluation of the model's predictive performance across different sequences in the updated version of the manuscript.

Point 3. Refine the description of the ablation experiments and highlight the conclusion that each component of the model's three architectures (CNN, LSTM, and Bi-LSTM) is meaningful.

Response: As per your valuable suggestion we have revised the ablation study and highlighted the conclusion in the updated version of the manuscript as:

"Ablation Study

In our ablation study, we conducted a comparative analysis to investigate the performance of hybrid deep learning models. The results, as detailed in Table 9 for each model. The CNN-LSTM model yielded an MSE of 0.095, RMSE of 0.309, MAE of 0.238, and MAPE of 31.84\%,

indicating a reasonable predictive performance. However, the CNN+Bi-LSTM model, with slightly higher error metrics, did not significantly outperform the CNN-LSTM. Notably, the BiLSTM+LSTM configuration achieved superior accuracy, with minimal errors across all metrics. This significant reduction in errors suggests that BiLSTM+LSTM is highly effective for this task, capturing complex patterns more accurately compared to the other configurations."

Table 9. The ablation study analysis.

Model	MSE	RMSE	MAE	MAPE
CNN-LSTM	0.095	0.309	0.238	31.84
CNN+Bi-LSTM	0.104	0.322	0.257	31.83
BiLSTM+LSTM	0.008	0.090	0.067	11.51

The conclusion is that each component of the model's three architectures:

2. Novel Layers fusion of model's architectures:

   Recent research separately used CNN, LSTM, and Bi-LSTM models, where our proposed hybrid model integrates three models for energy consumption prediction. This unique hybridization allows proposed model to capture spatial and temporal features of input data which make it possible to handle energy consumption behavior from different perspectives that might be ignored when a single model is employed.

3. Enhanced Feature Learning:

   CNN layer is deployed firstly in the architecture which allow hybrid model to extract local patterns and outliers in energy consumption data. This is usually most suitable for very short-term energy consumption prediction where the consumption pattern may quickly change. Recent studies focus on LSTM and Bi_LSTM for temporal prediction and neglecting the convolutional layers in feature extractions.

4. Bidirectional Processing:

   Bi-LSTM is important to improve accuracy and it has ability to capture both future and past contexts in the energy consumption time series dataset. In recent studies very rarely Bi-LSTM is integrated with CNN which provides a novel angel to our study.

4. Tackling Present Constraints and Other Obstacles:

   Proposed model overcome the limitations in existing literature such as inabilities to generalize across short-term and long-term energy consumption prediction simultaneously. Proposed model fills these gaps up contributes toward the existing body of knowledge in energy consumption prediction as a considerable addition.

In summary, according to the appraisal of the available contemporary resources on the subject, even though the proposed novel hybrid model features several components present in the literature, the combined use of CNN, LSTM, and Bi-LSTM for the task of energy consumption forecasting and its thorough testing on practical data is genuinely new and extends its usefulness within real-world situations. We feel these revolutions ought to be looked into more thoroughly and stress upon the relevance of our study towards the improvement of energy prediction methods and its relevance to the existing trends.

Point 4. Add an evaluation of the model's operational efficiency or computational complexity.

Response: As per your valuable suggestion we have added the computational complexity in the updated version of the manuscript as:

"Computational complexity

The computational complexity of the proposed model in Table 9 demonstrates its efficiency across varying temporal granularities. Using the IHEPC dataset, the model required 486 seconds for daily data, 119 seconds for weekly data, and just 32 seconds for hourly data. This performance highlights the model's scalability and suitability for both coarse-grained and fine-grained energy consumption analysis, ensuring practical usability across diverse applications."

Table 9. The computational complexity of the proposed model.

Data Portions	Runtime Computations (Seconds)
For Daily Data	486s
For Weekly Data	119s
For Hourly Data	32s

Point 5. Enlarge the font in the image results, and provide explanations for any special symbols.

Response: As per your valuable suggestion we have revised the image results in the updated version of the manuscript. In addition, for special symbols, we have added a subsection to the manuscript as:

"Acronyms

The acronyms in Table 10 are provided for clarity and convenience. These acronyms are intended to facilitate clear communication in the discussion of the proposed model, related work, and evaluation metrics."

Table 10. Description of acronyms used in this manuscript.

Short Name	Abbreviation
EECP	Electric Energy Consumption Prediction
CNN	Convolutional Neural Network
Bi-LSTM	Bi-directional Long Short-Term Memory
IHEPC	Individual Household Electric Power Consumption dataset
LSTM	Long Short-Term Memory
RNN	Recurrent Neural Network
MSE	Mean Square Error
RMSE	Root Mean Square Error
MAE	Mean Absolute Error
MAPE	Mean Absolute Percentage Error
MLP	Multilayer Perceptron
ReLU	Rectified Linear Unit
EECP-CBL	Electric Energy Consumption Prediction model utilizing the combination of CNN and Bi-LSTM
RF	Random forest
RT	Random Tree
CFS	Correlation-based feature selection
NCA FS	Neighborhood Component Analysis Regression-based feature selection
MRE	Mean relative error
SMAPE	Symmetric mean absolute percentage error
EDA	Exploratory data analysis
IMFs	Intrinsic mode functions
MLR	Multiple liner regression
BPNN	Back-propagation neural networks
EDL	Extreme deep learning
GBR	Gradient boosting regression
IDT	Information diffusion technology
HMTD	Heuristic mega-trend diffusion
LSTM-AE	Auto encoder long short-term memory
GRU	Gated-recurrent unit
SD	Standard deviation
EMD	Empirical mode decompositions
FFANN	Feedforward artificial neural network

Point 6. Notably, the performance of the model proposed by the authors, especially in terms of MSE, was hundreds of times lower than that of other common time series prediction models. If there are no issues, such as recording errors, in the experimental results, this would represent a significant breakthrough. It is recommended that the authors submit their code to verify the authenticity of the experimental results.

Response: The authors are highly grateful for your efforts and insightful comments. As per your valuable suggestion, we have uploaded the coding experiments with this revision to the PLOS One system.

Point 7. In summary, the ideas in this paper are highly creative, and the structure is relatively clear. If the authors agree with and address the issues mentioned above, I recommend it for publication.

Response: The authors are highly grateful for your efforts and insightful comments. The paper is extensively revised to remove ambiguity as per your valuable suggestions.
